# Modern Sociology

**Published:** 1899-02-04

**Authors:** 


### Modern Sociology.

### THE ANOMALY OF THE HALF-TIMER.

When, some time ago, we spoke in the pages of The Hospital of the great improvement in the condition of the children of the industrial classes that has taken place during the present reign, it was admitted that the existence of the half-timer was a survival of an old and false notion of the relation of parent and child which was much to be regretted. The concession which permitted children above the age of ten to work for half the week, and go to school the other, was intended only for the relief of poor parents. But who shall say who is poor? As a matter of fact, the permission has been taken advantage of by all parents, or nearly all, in those districts where child labour used to be rampant, and where the tradition of it has not yet been forgotten. Now, however, it is not the manufacturers who want children in their mills. It is the parents who are to blame if this anomaly survives to the present day; and the sooner the parent of the industrial class is taught that it is his duty to keep his child till he is fairly well grown and well educated, and not his child's duty to keep him, the better it will be for the British nation.

A commissioner from the *Daily News* has been going round the districts in Lancashire where the great textile factories are, and has amassed an amount of information which must impress everyone who has the welfare of the working classes at heart. In his investigations the *Daily News* has had the advantage of the assistance of Dr. Torrop, of Heywood, who is known to the present writer as a gentleman of high intelligence and principle, and who has the experience gained by having worked for thirty-three years in the place he is speaking of. The statement he makes, added to the statistics collected by the representative of the *Daily News*, show conclusively that the half-timer, especially if he is the child of parents who have since their childhood been working in spinning or weaving mills, is a poor and stunted being, infinitely inferior in health, strength, and it might be added intelligence, to an ordinary child of his age.

The people of Lancashire are, as a rule, a strong, large-built, healthy race; vigorous, clear-complexioned, and comely. That is what they are out on the moors. But in the industrial towns they are none of these things, being poor, stunted under-sized creatures. This is due partly to conditions that are now past, when factories were as unhealthy as possible, But it is due partly, also, to the vicious system which, under the pretext of helping poor parents, allows children who are not yet done growing, and have not yet assimilated the rudiments of education, to be put to work in the mills. It is doubtful if the half-timer is better educated under the present system than he would be if he left school entirely when he entered the mill. The strain of alternate school and work probably ends in his doing but little good at either. He would certainly prove in the end a more intelligent and valuable workman, if he went on for a year or two longer devoting himself entirely to study and play. Physically he is markedly degenerate. As Dr. Torrop puts it: "The promising children of 10 degenerate into the lean and sallow young persons of 13, and so the process continues as they grow older, until a whole population becomes stunted, and thus the conditions of life in a factory become a real source of danger to England's future."

Factory life at its best does not make for health. Build it as well as you may, study its sanitation as carefully, and have its management supervised as rigidly as possible, you cannot make factory work as healthful as, on the whole, farm work is; this we must accept. If, then, we are unwilling to return to the primitive life of Eden, what are we to do? Simply to allow the animal development to go on as far as possible before any labour is undertaken which will interfere with it. A man or woman who has a strong healthy physique to begin with will go through a strain which would kill one of weaklier build, and that without appreciable deterioration. But at ten years of age no child is fit for any severe strain, either of mind or body, nor is he able to throw off the ill-effects of confinement and bad air. Yet the half-time system permits a ten-year-old child to undergo a double strain, physical and intellectual, which would try a full-grown man. Teachers will tell any inquirer how little profit half-timers get from their lessons. A few years after going to work many of them are hardly able to spell out a page of newspaper, or to write their own names. The elaborate machinery of education which the nation provides is almost wasted on them. Doctors will tell how stunted their frames grow. Dr. Torrop says that the average weight of factory children is 18 lb. below the average of the whole of England. They suffer to an exceptional extent from headache, induced by the heat and oily smell of the weaving sheds. Toothache also is common, caused by the hot humid air; and eye complaints, chronic neuralgia, scrofula, with its developments in consumption, hip disease, rickets, and the like, are more common than they are in other conditions. And finally, employers will tell you that, as workers, half-timers are worth but little. The old love for cheap labour is fast vanishing. Quality for quality, well-paid and intelligent labour can always compete successfully with ill-paid and unintelligent labour. Witness the number of things which America, where the working-man is better paid, better housed, and better fed than anywhere else on earth, can supply us with cheaper than we can produce them ourselves. Is the case of Germany, said to be cutting us out in so many markets, to be quoted against this statement? Impartial observers are beginning to see that Germany is purchasing a temporary advantage at the cost of the health of her people, who are becoming undersized and anæmic, lacking in energy and initiative. Besides, we said, "quality for quality" and "made in Germany" is, as everyone knows, a synonym for shoddy. We are more likely to be able to compete with Germany and every other country by keeping our children longer at school, if the school is one where their faculties are trained as well as their brains crammed, and by encouraging them to play, than by robbing them of their birth-right of fresh air, and setting them to help maintain their parents, by this cruel survival of old sins and follies, the "half-time" system.

